# Synthetic Thioesters of Thiamine: Promising Tools for Slowing Progression of Neurodegenerative Diseases †

**DOI:** 10.3390/ijms241411296

**Published:** 2023-07-10

**Authors:** Lucien Bettendorff

**Affiliations:** Laboratory of Neurophysiology, GIGA Neurosciences, University of Liège, 4000 Liège, Belgium; l.bettendorff@uliege.be; Tel.: +32-4-366-5967

**Keywords:** benfotiamine, O,S-dibenzoylthiamine, Alzheimer’s disease, oxidative stress, neuro-inflammation, amyotrophic lateral sclerosis

## Abstract

Thiamine (vitamin B1) is essential for the brain. This is attributed to the coenzyme role of thiamine diphosphate (ThDP) in glucose and energy metabolism. The synthetic thiamine prodrug, the thioester benfotiamine (BFT), has been extensively studied and has beneficial effects both in rodent models of neurodegeneration and in human clinical studies. BFT has no known adverse effects and improves cognitive outcomes in patients with mild Alzheimer’s disease. In cell culture and animal models, BFT has antioxidant and anti-inflammatory properties that seem to be mediated by a mechanism independent of the coenzyme function of ThDP. Recent in vitro studies show that another thiamine thioester, O,S-dibenzoylthiamine (DBT), is even more efficient than BFT, especially with respect to its anti-inflammatory potency, and is effective at lower concentrations. Thiamine thioesters have pleiotropic properties linked to an increase in circulating thiamine concentrations and possibly in hitherto unidentified open thiazole ring derivatives. The identification of the active neuroprotective metabolites and the clarification of their mechanism of action open extremely promising perspectives in the field of neurodegenerative, neurodevelopmental, and psychiatric conditions. The present review aims to summarize existing data on the neuroprotective effects of thiamine thioesters and give a comprehensive account.

## 1. Introduction

**Figure 1:**
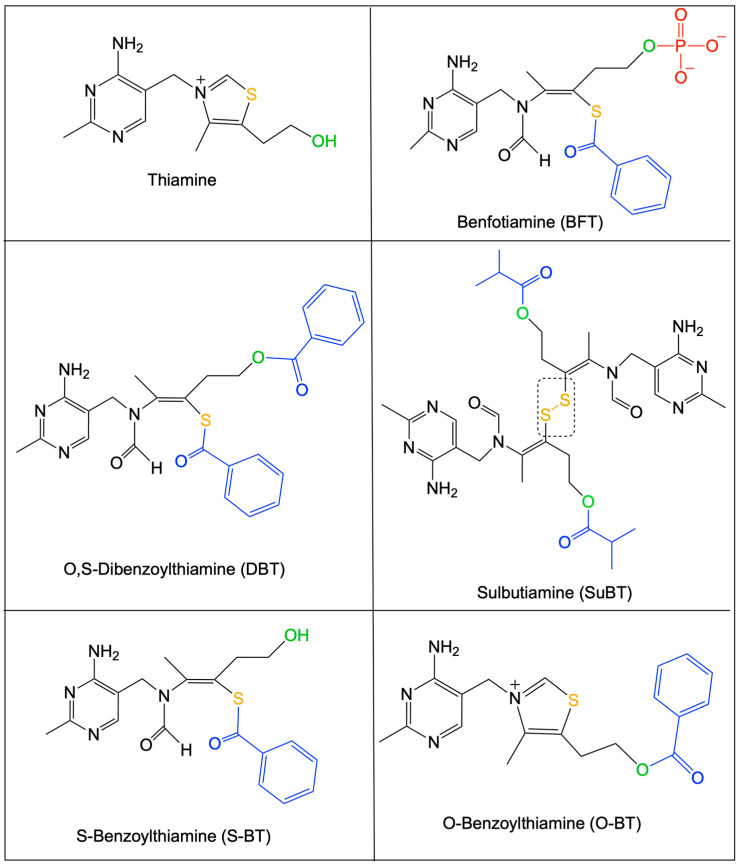
Structural formulas of thiamine and thiamine precursors (prodrugs) with high bioavailability. The sulfur atom of the thiamine thiazolium ring is shown in yellow. The alcohol group of thiamine is shown in green. Organic substituents are in blue, and the phosphate group of BFT is shown in red. SuBT (a symmetric dimer) is a disulfide, while BFT, DBT, and S-BT are thioesters. O-Benzoylthiamine (O-BT) results from the hydrolysis of the thioester in DBT or through an intramolecular rearrangement of S-BT followed by spontaneous thiazole ring closure [10]. Note that for all the open thiazole ring derivatives, the -CH_3_ group and the sulfur group must be in the “trans” position to allow thiamine to be formed [12]. This is the case for the (Z)-isomer of BFT shown here and which is the only one that should be called “benfotiamine”.

**Figure 2:**
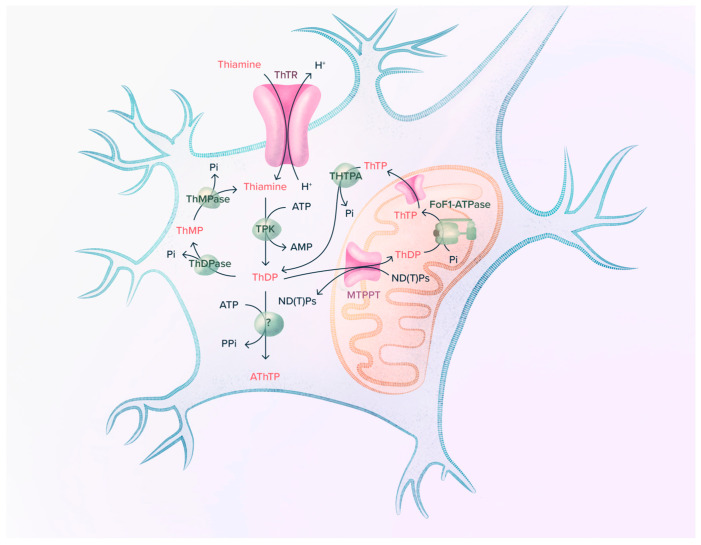
Thiamine metabolism in a neuron. Thiamine is transported into the cells and then pyrophosphorylated by thiamine pyrophosphokinase (TPK). This free ThDP of high turnover can either bind to transketolase or be transported into mitochondria, where it binds to 2-oxoacid dehydrogenase apoenzymes (see Figure 3). The sum of bound ThDP represents the low turnover pool [9]. Excess cytosolic ThDP is hydrolyzed to ThMP by thiamine pyrophosphatases (ThDPase), and ThMP is hydrolyzed to thiamine by thiamine monophosphatases (ThMPase). A small fraction of mitochondrial ThDP can be phosphorylated to ThTP by FoF1-ATP synthase. ThTP can exit mitochondria and be hydrolyzed to ThDP by a very specific cytosolic 25-kDa thiamine triphosphatase (THTPA) [18,19]. Cytosolic ThDP can also be converted to AThTP by a ThDP adenylyl transferase [20].

Thiamine (vitamin B1, Figure 1), probably the most pleiotropic of the B vitamins, is involved in many cellular processes (energy metabolism, cell survival, antioxidant activity …). Thiamine diphosphate (ThDP), a coenzyme for over 20 enzymes (among which are 6 enzymes and enzyme complexes in mammalian cells), is involved in cell energy metabolism as well as other metabolic functions (Figure 2) [1]. Other derivatives (Figure 1), among which thiamine triphosphate (ThTP, [2,3,4,5]) and adenosine thiamine triphosphate (AThTP, [6,7]), are thought to be involved in coenzyme-independent functions.

ThDP can be hydrolyzed to thiamine monophosphate (ThMP) by various phosphatases [8,9]. Whether ThMP has a specific physiological function remains unknown, but it seems to have some antinociceptive effects mediated by prostatic acid phosphatase [10].

We recommend using the abbreviations ThMP, ThDP, ThTP rather than TMP, TDP, or TTP in order to avoid confusion with phosphorylated thymidine derivatives (officially TMP, TDP, and TTP) as happened in the past [11].

Two recent clinical studies suggested that a thiamine prodrug, benfotiamine (BFT), might be helpful in slowing down the evolution of Alzheimer’s disease [13,14]. While many studies showed beneficial pharmacological effects of BFT in mouse models of neurodegenerative diseases [15,16,17], the mechanism of action is rarely discussed in terms of active metabolites and their molecular targets. In this review, after a brief introduction to the biochemical properties of thiamine and homeostasis, we will summarize the information available on the possible molecular targets of BFT and other thiamine prodrugs, as well as the possible metabolites involved. We clearly want to distinguish between physiological coenzyme effects and pharmacological, coenzyme-independent effects of thiamine prodrugs. With this respect, we will also examine the limitations and pitfalls of animal and cell culture models and the information that can be obtained.

**Figure 3:**
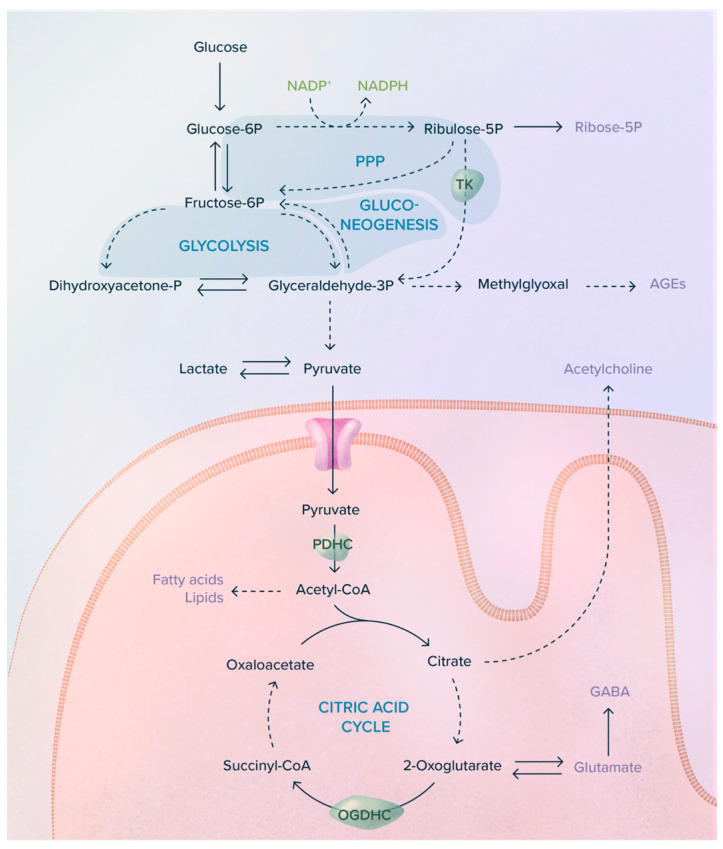
Role of ThDP-dependent enzymes in cell metabolism. TK, transketolase; PDHC, pyruvate dehydrogenase complex; OGDHC, 2-oxoglutarate dehydrogenase complex. The mitochondrial PDHC-catalyzed reaction is an important source of acetyl-CoA, the precursor of the neurotransmitter acetylcholine, via citrate as an intermediate [21]. Broken lines represent steps involving several reactions.

## 2. Transport and Metabolism of Thiamine Derivatives

Biosynthesis of thiamine, and especially the thiazole moiety, is complex and slightly differs among bacteria, yeast, and plants [22,23,24]. As there are no thiamine synthesizing pathways in animals, thiamine biosynthesis will not be discussed here.

In humans, the vast amount of thiamine is of alimentary origin. After transport across the intestinal epithelium, thiamine enters the brain via two transporters of the folate/thiamine transporter family: *SLC19A2* (thiamine transporter 1, ThTR1) and *SLC19A3* (thiamine transporter 2, ThTR2). These high-affinity transporters function as thiamine/H^+^ antiporters [25,26,27]. The *K*_m_ for thiamine is 2.5 ± 0.6 µM for ThTR1 [28] and 27 ± 8 nM for ThTR2 [29]. While these transporters are well characterized from a molecular point of view, their distribution in the human brain remains poorly understood, and to our knowledge, only one study partially addressed this issue [30]. According to this study, ThTR1 and ThTR2 are expressed in the blood vessels throughout the brain, with ThTR1 immunoreactivity present only at the luminal side of the blood vessels, while ThTR2 is expressed at the basement membrane and in perivascular pericytes. Hence, both types of transporters may be involved in thiamine transport across the blood–brain barrier. In the human brain, ThTR2 seems to have a higher expression pattern in neurons than ThTR1 [30].

Inside cells, thiamine is pyrophosphorylated to ThDP by thiamine pyrophosphokinase (TPK) [31,32]. The main fates of ThDP are binding to apotransketolase or transport into mitochondria [8,9]. ThDP is transported into mitochondria by a specific carrier (the mitochondrial thiamine pyrophosphate transporter, MTPPT), probably in exchange for nucleoside di- or tri-phosphates [33] (Figure 2). ThDP can be hydrolyzed to ThMP, which in turn can be hydrolyzed to thiamine by various phosphatases, including prostatic acid phosphatase [1,10]. Extracellular ThMP probably originates from cellular efflux through the reduced folate transporter 1 [34].

Triphosphorylated thiamine derivatives exist in most cells (for a discussion, see [4], Figure 2). ThTP is synthesized in brain mitochondria by oxidative phosphorylation, probably involving F_O_F_1_-ATP synthase [35]. AThTP, probably synthesized by a cytosolic thiamine diphosphate adenylyl transferase [7,20], exists in small amounts in mammalian tissues [36]. However, both compounds are probably not involved in the phenomena described here and will not be discussed further.

## 3. Coenzyme Function of Thiamine Diphosphate

### 3.1. ThDP-Dependent Enzymes in Mammals

ThDP is the coenzyme for many enzymes and enzyme complexes involved in carbohydrate and amino acid catabolism. In mammals, the most important are pyruvate (PDH, EC 1.2.4.1) and 2-oxoglutarate (OGDH, EC 1.2.4.2) dehydrogenases that catalyze two essential steps in citric acid metabolism ([26], Figure 3) and transketolase (TK, EC 2.2.1.1) in the pentose phosphate pathway (PPP). Hence, ThDP-dependent enzymes PDH and OGDH are responsible for nearly two-thirds of the CO_2_ released during the complete oxidation of glucose. Indeed, only about 5% of glucose is metabolized through the PPP in the brain [37].

As these enzymes catalyze essential steps in glucose oxidation, it is obvious that ThDP is an indispensable coenzyme for energy metabolism, and it is not surprising that thiamine deficiency will have deleterious effects on organs that are particularly dependent on oxidative metabolism, such as the nervous and cardiovascular systems. In addition to catalyzing essential reactions in cell energy metabolism [38], PDH and OGDH are also important for the synthesis of the neurotransmitters glutamate, GABA, and acetylcholine [39].

TK is a key rate-limiting enzyme of the PPP [40]. This pathway is essential for the synthesis of (1) NADPH, which is important for reductive biosynthesis and protection against oxidative stress, and (2) ribose 5-phosphate.

In addition, ThDP is a coenzyme for at least three other mammalian enzymes [41]:

- Mitochondrial oxoacid dehydrogenase, involved in lysine-derived 2-oxoadipate catabolism [42,43];
- Mitochondrial branched-chain 2-oxoacid dehydrogenase (EC 1.2.4.4) [44] is required for the catabolism of branched-chain amino acids;
- Peroxisomal 2-hydroxyacyl-CoA lyase (EC 4.1.2.63) in α-oxidation of phytanoic acids [38].

However, the implication of the latter enzymes in the symptoms of thiamine deficiency disorders is less documented [26].

### 3.2. ThDP Mediated Catalysis

**Figure 4:**
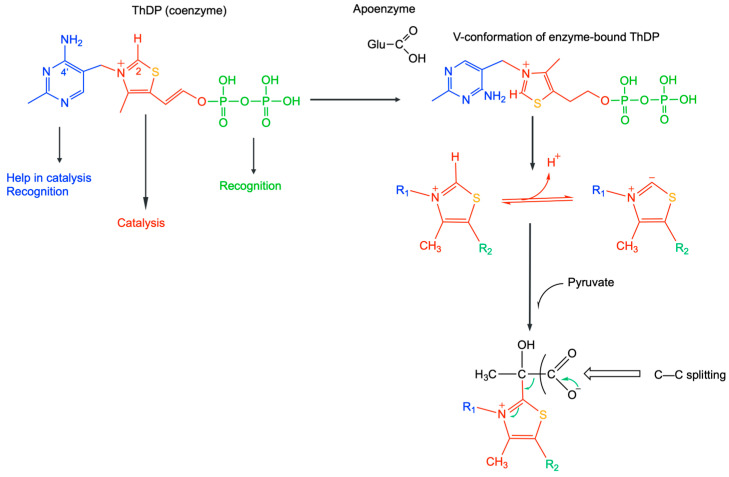
Mechanism of thiamine diphosphate catalysis. The apoenzymes force the thiamine molecule in the so-called V-conformation bringing the 4′-amino group close to the thiazolium C-2 proton, favoring deprotonation. The catalytic action of ThDP is linked to the ability of its thiazolium ring to form an ylide (carbanion) intermediate by deprotonation of the thiazolium C-2 and attack of the resulting carbanion on a substrate (here shown for pyruvate) carbonyl.

The ThDP molecule is composed of three parts: the catalytically active thiazole heterocycle, the pyrimidine ring, and the phosphorylated hydroxyethyl chain (Figure 4). Ronald Breslow was the first to propose that the thiazolium ring is the core element for catalysis by ThDP [45,46]. Though the pyrimidine ring of ThDP is not essential for catalysis, a conserved Glu residue in ThDP-dependent enzymes facilitates the adoption of the so-called V-conformation, forcing an angle between the thiazolium and pyrimidine rings in such a way as to bring closer the 4′-amino group (as an imino tautomer) and the thiazolium ring in order to favor deprotonation of the C-2 proton. The diphosphate group plays no role in catalysis and is only required for tight binding of ThDP to the apoenzymes.

In mammalian enzymes, ThDP catalyzes the cleavage of C-C bonds immediately adjacent to a carbonyl group (α cleavage). Indeed, cleavage of a C-C bond requires stabilization of the product, which is immediate for β cleavage but not for α cleavage, thus requiring an additional tool brought by the thiamine thiazole ring [47].

## 4. Thiamine Status in Humans and Inter-Organ Homeostasis

Thiamine status in humans is an important issue in view of the high number of populations at risk of thiamine deficiency around the world and, in particular, in children [48,49]. The clinical symptoms of thiamine deficiency syndromes are highly variable and affect the cardiovascular and nervous systems. Typical thiamine deficiency syndromes are beriberi and Wernicke’s encephalopathy. Both are highly reversible after administration of thiamine [50]. In addition to the severe forms of thiamine deficiency, subclinical thiamine deficiency can be relatively common in humans.

### 4.1. Thiamine Status in Humans

One of the central questions is to understand the distribution of thiamine derivatives between different organs. In rats, only 1.5% of total thiamine is found in the brain, while 22% is found in the liver [51]. Total thiamine levels are three to four times lower in the brain than in the liver [36]. More significantly, during thiamine deficiency, thiamine levels decrease less steeply in the brain than in the liver [51,52]. The picture that is emerging from rodent models suggests that when thiamine (or thiamine prodrugs) is administered, it is captured and phosphorylated in the liver and, to a lesser extent, in erythrocytes both compartments acting as transient buffers of blood thiamine levels. When thiamine levels normalize again, mainly through renal excretion, hepatocytes, and erythrocytes release excess thiamine, which is then eliminated. In contrast, brain thiamine levels are highly regulated such as to minimize variations in thiamine and thiamine phosphate levels in either direction.

In humans, we have only access to circulating thiamine levels, and it is not clear to what extent they reflect brain thiamine (and ThDP) levels. Indeed, we do not know how brain thiamine levels are regulated in humans, but there is no reason to assume that the mechanisms are different from those in rodents.

Therefore, an important issue is to know how much brain ThDP levels must decrease before overt symptoms of thiamine deficiency appear. In rats, brain thiamine levels must drop to about 10–20% of normal before symptoms of thiamine deficiency appear [36,52,53]. Expressed in pmol/mg, this is not much lower than normal levels in the human brain (Table 1). If we assume that ThDP requirements are not different between humans and rodents, then both circulating and brain ThDP levels in healthy humans are close to the threshold of deficiency (Table 1). This would explain that a subclinical thiamine status in humans is relatively frequent [49].

Hence, it seems that erythrocyte and liver thiamine content is more or less proportional to the plasma thiamine concentrations, while brain thiamine content is much more tightly controlled. The latter is only affected when plasma thiamine concentrations are decreased over an extended period during a chronic thiamine deficiency period.

Two features may play an important role in brain thiamine homeostasis: transport across the BBB and regulation of ThDP synthesis.

### 4.2. Thiamine Transport across the Blood–Brain Barrier Is Limited by Trans-Stimulation

When loading cultured mouse neuroblastoma cells with ^14^C-thiamine, we observed that after eliminating the extracellular tracer, the cells released ^14^C-thiamine into the extracellular medium. This release was stimulated by extracellular thiamine in the 0.1–1 µM range [60].

Years later, using an in situ rat brain perfusion method, it was demonstrated that thiamine efflux through the BBB was also trans-stimulated by thiamine in the sub-micromolar range [61]. Plasma thiamine concentrations are approximately 10 nM in humans (Table 1) and 100 nM in rodents. A trans-stimulation would occur when the plasma thiamine concentration is increased above 100 nM, which is easily attained in humans after administration of thiamine prodrugs.

**Figure 5:**
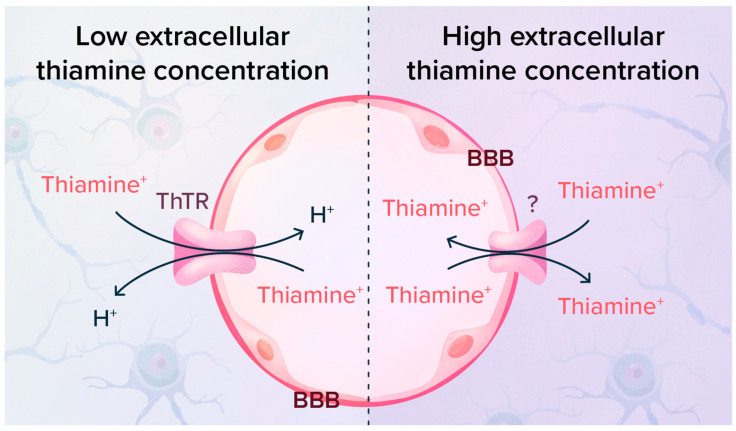
High extracellular thiamine concentrations favor a thiamine^+^/thiamine^+^ over a thiamine^+^/H^+^ exchange mode across the BBB [61]. Such a trans-stimulation was also observed in cultured neuroblastoma cells [60]. It is not clear whether the same transport protein is involved in the two modes and which compartment of the BBB would be involved in such behavior.

The above data suggest that a mechanism protecting the brain from a thiamine overload is operating through a thiamine/thiamine exchange. It is not clear whether the known transporters can switch from a thiamine^+^/H^+^ to a thiamine^+^/thiamine^+^ exchange mode (Figure 5) or whether a specific protein, different from ThTR1 or ThTR2, mediates thiamine efflux.

### 4.3. TPK Is Regulated by Retroinhibition of ThDP

We observed numerous times that in animals treated with thiamine or BFT, blood thiamine levels may increase by several orders of magnitude, but ThDP levels are only slightly increased [56]. A similar observation was made in cultured cells exposed to BFT, suggesting a very tight control of ThDP synthesis [9,56].

**Figure 6:**
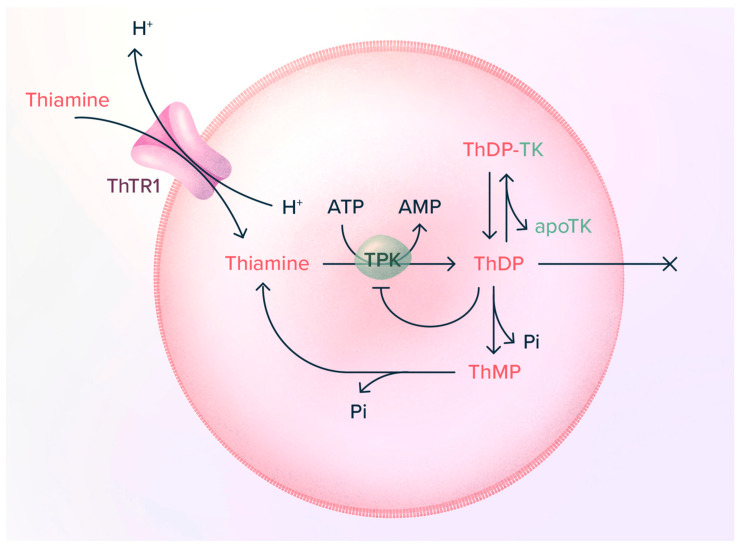
Regulation of ThDP synthesis by TPK in a red blood cell. Thiamine is transported into the cell by a thiamine^+^/H^+^ exchange, probably involving ThTR1 [62]. Inside the cells, thiamine is pyrophosphorylated by TPK to ThDP, which acts as a feedback inhibitor of TPK when intracellular thiamine concentration exceeds 0.5 µM. ThDP cannot exist across the plasma membrane by simple diffusion. ThDP binds to apotransketolase, and excess coenzyme is hydrolyzed to ThMP and thiamine. Note that in physiological solutions, the positive charge of thiamine is neutralized by a chloride anion.

Indeed, when studying the kinetics of recombinant mouse TPK in the forward (physiological) direction (thiamine + ATP ⇆ ThDP + AMP) (Figure 2 and Figure 6), we observed that the reaction stopped even when only a small proportion of thiamine had been converted to ThDP [32]. This could be explained by the observation that ThDP is a potent non-competitive inhibitor of TPK with a *K*_i_ of approximately 0.2–0.4 µM, of the same order of magnitude as free intracellular ThDP concentrations. This observation may explain why, even with a large excess of thiamine, ThDP does not accumulate within cells (Figure 6).

A possible justification for such behavior would be the protection of the brain from thiamine depletion. Indeed, if thiamine could be phosphorylated to ThDP without limit (the equilibrium of the reaction is strongly towards ThDP synthesis [32]), this could lead to a capture of plasma thiamine by red blood cells and hepatocytes (the latter having a high capacity transporter for thiamine, see Section 6.1) and depletion of the plasma thiamine pool, decreasing its availability for the brain [32].

## 5. Development of Synthetic Thiamine Derivatives with High Bioavailability

Thiamine transport across cell membranes is a relatively slow process [27]; therefore, several synthetic thiamine prodrugs with high bioavailability were developed, mainly with the aim of rapidly restoring brain thiamine levels after deficiency. These derivatives have in common that they are relatively hydrophobic, or they generate hydrophobic metabolites allowing a rapid diffusion across the intestinal permeability barrier [26].

**Figure 7:**
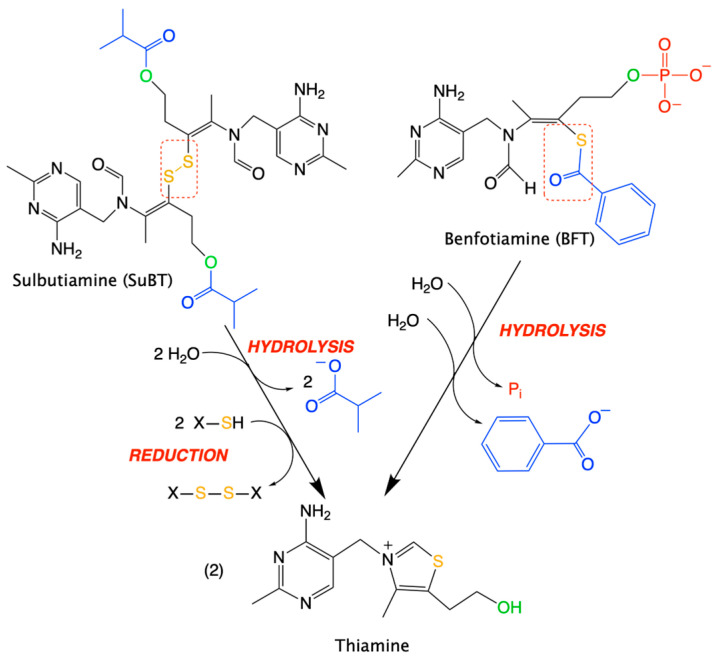
Sequences of reactions leading from SuBT (hydrolysis + reduction) or BFT (2 × hydrolysis) to thiamine. Please note that with the symmetric dimer SuBT, two molecules of thiamine are formed. Here, we suppose that the cleavage of the disulfide link in SuBT occurs via reduction by another thiol, the most obvious being reduced glutathione.

We must distinguish between two chemically distinct types of prodrugs (Figure 7):

1. Disulfides, among which the most prominent representative is SuBT and its product of hydrolysis thiamine disulfide [63];
2. Thioesters, represented by BFT [56,64].

Both types of molecules have an open thiazole ring, but the distinction between the two groups is important as their metabolization requires different reactions to form thiamine: disulfides require reduction of the disulfide bond, while thioesters require hydrolysis.

## 6. BFT in Animal Studies

### 6.1. Administration of BFT in Mice Does Not Significantly Increase Brain ThDP Levels

There are only a few systematic pharmacokinetic studies with BFT, and those existing limit themselves to the determination of thiamine, ThMP, and ThDP in blood for the estimation of its bioavailability [65,66]. To our knowledge, no attempt has been made to detect BFT or potential byproducts (S-BT or O-BT, for instance, Figure 1). Among the many studies that tested the potential therapeutic properties of BFT, rare are those that tested their effect on levels of thiamine derivatives in the target organ (see [67], for instance). As late as 2007, no data on the effects of BFT on nervous tissue were available, and so it became overdue to check the effect of this molecule on brain thiamine levels.

**Figure 8:**
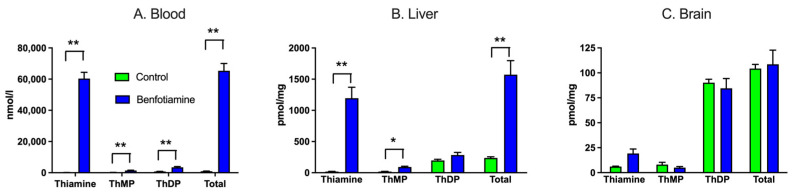
Thiamine, ThMP, and ThDP levels one hour after a single oral administration of benfotiamine (100 mg/kg) or control solution. The data were analyzed by MANOVA (Wilk’s Lambda, *p* = 0.0002, 0.0145, and 0.27 for blood, liver, and brain, respectively) followed by ANOVA for each thiamine compound (*, *p* < 0.05; **, *p* < 0.01). The results are expressed as mean ± SEM for 4 animals in each group. (Modified according to [56]).

Surprisingly, the administration of high levels of BFT increased total thiamine in the blood and liver, but not in the brain (Figure 8, [56]). The absence of the effect of BFT on brain thiamine levels, though it strongly increases circulating thiamine levels, can be explained by the above-mentioned trans-stimulation of thiamine uptake across the BBB.

In addition, while thiamine concentrations increased by two or three orders of magnitude above control levels in blood and liver, ThDP concentrations were only marginally increased, in agreement with a feedback inhibition of TPK by ThDP [32] (Figure 6).

A third factor might be a slower thiamine transport across the BBB than into hepatocytes. Indeed, in rats, radioactive thiamine entered the liver much more rapidly than the brain [68], which would suggest the involvement, in the liver, of a high-capacity thiamine transporter different from ThTR1 or ThTR2 (which are dependent on the H^+^-gradient). It has been suggested that the organic cation transporter 1 might be involved in thiamine uptake by hepatocytes [69,70,71]. It was also claimed that thiamine uptake in isolated rat hepatocytes is Na^+^-dependent [72,73], but this is probably rather a dependence on the Na^+^ gradient rather than Na^+^ itself [27]. Organic cation transporters are Na^+^-independent, but their electrogenic nature makes them indirectly dependent on the Na^+^ gradient via the membrane potential [69].

### 6.2. BFT Has Beneficial Effects in Animal Models of Neurodegenerative Diseases, Stress, and Anxiety

Paradoxically, in addition to the intriguing observation that BFT has apparently no significant effect on brain thiamine content, many studies document neurological effects of BFT while, at the same time, reporting no increase in brain ThDP levels, suggesting that the coenzyme function of ThDP is not involved.

For instance, Pan et al. (2010) [25], in a landmark study, investigated the effects of chronic treatment by BFT in amyloid precursor protein/presenilin (APP/PS1) double transgenic mice, a classical model of Alzheimer’s disease. Oral treatment (100–200 mg/kg/day) for 8 weeks enhanced spatial memory and reduced amyloid plaque numbers and phosphorylated tau levels in cortical areas. Remarkably, no such beneficial effects were seen when the mice were treated with the thiamine prodrug fursultiamine suggesting a certain specificity for BFT.

More recently, a similar treatment with high doses of BFT (200 mg/kg per day for 8 weeks) was applied in a mouse model of tauopathy, P301S mice [26]. In this model, BFT increased lifespan, prevented the death of spinal neurons, and improved behavioral deficits. It also decreased oxidative stress, inflammation, accumulation of advanced glycation end products (AGEs), tau phosphorylation, and formation of neurofibrillary tangles in the cerebral cortex and hippocampus. 

In addition to these therapeutic actions in mouse models of neurodegeneration, BFT treatment counteracted anxiety and depression-like behavior as well as aggression linked to emotional stress. Stress was induced either by a modified swim test, the presence of a predator for five consecutive nights [48,49], or chronic ultrasound exposure for 20 days [50].

BFT also normalized plasticity markers, stimulated neurogenesis, and improved memory:

1. The administration of BFT decreased aggression, reversed ultrasound-induced changes in GluA1 and GluA2 subunit expression, and reversed the decreased expression of plasticity markers [74];
2. BFT (more efficiently than thiamine) counteracted the predator stress-induced decrease in the proliferation and survival of newborn immature neurons in the subgranular zone of the dentate gyrus [75].

These data suggest that BFT may have a therapeutic potential not only in neurodegenerative diseases but also in other brain pathologies, such as major depression linked to stressful events. At least some of these effects may be mediated by GSK-3β and possibly GSK-3α downregulation [16,76]. Changes in expression and phosphorylation of GSK3β were reported in mouse models of stress-induced anxiety and depression [76]. In the prefrontal cortex, mRNA levels of GSK3β were increased in the modified swim test and when the mice were exposed to predator stress. This increase was fully reversed when the animals were treated with BFT.

### 6.3. BFT Has Coenzyme-Independent Antioxidant and Anti-Inflammatory Properties

It is essential to know the mechanism by which BFT exerts these various effects.

In all the cited experiments, the mice received a normal chow, rich in thiamine, and we can suppose that all ThDP-dependent enzymes were saturated by the coenzyme ThDP. Moreover, treatment with BFT did not increase brain ThDP levels [15,16,56,75]. Hence, the observed effects are probably coenzyme independent.

According to Tapias et al., BFT activates the Nrf2/antioxidant responsive element (ARE) [15]. However, the activation of this pathway seems to require relatively high BFT concentrations (>100 µM) in cultured cells [77]. As such high concentrations of either BFT or one of its metabolites are unlikely to be reached in the brain parenchyma, and Nrf2 is unlikely to be a direct target of thiamine prodrugs in vivo. However, it is possible that the NrF2 antioxidant pathway is indirectly activated.

Gorlova et al. report a normalization of brain oxidative stress markers by BFT after ultrasound-induced aggression [74]. They suggest that this effect may be due to decreased GSK-3β activity, ultimately impacting Nrf2 signaling. Indeed GSK-3β phosphorylates Nrf2, resulting in its nuclear exclusion [78]. In this hypothesis, the effect of BFT would be on GSK-3β, or on a factor upstream of GSK-3β rather than Nrf2. Such a hypothesis would explain the activation of Nrf2 target genes by BFT in vivo [15].

BFT also has strong antioxidant effects in neuroblastoma cells [77] and BV2 microglial cells, probably targeting glutathione metabolism [79].

Microglial cells are resident macrophage-like immune cells of the nervous system. The overactivation of microglia induces the production of neurotoxic reactive oxygen and nitrogen species. This process is likely to play an important role in neurodegenerative processes [80].

In addition to antioxidant effects, BFT also significantly reduced anti-inflammatory markers in vivo, among which reduced iNOS expression and NF-κB p65 immunoreactivity [15,75,81]. BFT counteracts the morphological changes corresponding to the LPS-induced activation of the microglial cells. In addition, it decreases the production of proinflammatory mediators such as iNOS, COX-2, TNF-α, and IL6. These effects of BFT are likely mediated by the suppression of NF-κB translocation to the nucleus [82].

## 7. BFT in Human Clinical Studies

### 7.1. Thiamine Homeostasis Is Disrupted in Patients with Alzheimer’s Disease

There are only a handful of studies on thiamine status in Alzheimer’s patients, but they consistently show a lower status compared to age-matched controls. Indeed, several independent studies have reported abnormalities of thiamine homeostasis in patients suffering from neurodegenerative diseases. An early study reported a 20% decrease in ThDP levels in postmortem brains of patients with Alzheimer’s disease compared with adequately matched controls [83]. Moreover, a 40–50% decrease in postmortem ThDP levels was observed in patients with frontal lobe degeneration of the non-Alzheimer type [84], but in this study, the number of patients was small (n = 6). Much more recently, it was shown that patients with Alzheimer’s disease had 20–30% decreased blood ThDP concentrations compared to controls, while ThMP and thiamine levels were not impaired [85,86]. The same authors reported increased ThDPase and ThMPase activities which might contribute to the reduction of ThDP concentrations [87]. They also showed that ThDP reduction strongly correlates with brain glucose hypometabolism in Alzheimer’s disease [88], suggesting that a “subclinical” thiamine deficiency (i.e., not sufficient to produce overt symptoms of thiamine deficiency such as Wernicke’s encephalopathy, but nevertheless significant) might contribute to the clinical manifestations of Alzheimer’s disease.

It is to be expected that such a subclinical deficiency would be immediately corrected by the administration of thiamine or thiamine precursors. Indeed, it is well-known that thiamine deficiency disorders are readily reversible [49].

It is not clear why thiamine homeostasis is impaired in Alzheimer’s disease patients, but an explanation may come from a recent study showing a significantly lower expression of both THTR-1 and THTR-2 in the brains of Alzheimer’s disease patients leading to lower brain thiamine content [89]. The authors suggest that proinflammatory cytokines impair thiamine transport in these patients.

### 7.2. Therapeutic Effects of BFT on Patients with Alzheimer’s Disease

The first study testing BFT (300 mg daily over 18 months) in Alzheimer’s disease patients was carried out by the group of Chunjiu Zhong at Fudan University in Shanghai. The study was conducted on a relatively small sample of mild to moderate Alzheimer’s disease patients (n = 5) [13]. BFT significantly improved the cognitive abilities of the patients independently of brain amyloid accumulation.

A second study on a larger sample of patients (n = 34) was conducted by the group of Gary Gibson at the Burke Neurological Institute in New York [14]. BFT (2 × 300 mg for 12 months) slowed down cognitive decline compared to the placebo group, as viewed by the clinical dementia rating, and this effect was stronger in the *APOE* 4 non-carriers. Furthermore, BFT also significantly reduced increases in blood AGE charge. Serum metabolic and lipid profiling on a small subset of these patients showed that BFT normalized several amino acids (Tyr, Trp, His) and lipids (phosphatidylcholine) to levels observed in the control placebo group [90].

The results obtained in these clinical studies, though not conclusive, are indeed very promising. An important point concerns the doses used in both studies. In mice, the effects of BFT were much more spectacular than in the two human studies, but the doses used were much higher (200 mg/kg per day). Extrapolated to humans, such a dose would correspond to approximately 14 g of BFT per day. This could, of course, be one of the reasons for the differences observed between animal and human studies.

A further point requiring attention is that Alzheimer’s disease patients have lower brain and blood thiamine levels (see Section 7.1), a situation not yet reported in animal models [15,16]. The reason is probably that experimental animals are kept on a thiamine-rich chow, compensating for any effects of the pathology on thiamine transport. Therefore, we must expect that BFT administration in humans, in addition to its pharmacological effects, will correct thiamine levels.

## 8. O,S-Dibenzoylthiamine, a Promising New Thiamine Prodrug

In view of the above-mentioned considerations, it should be of interest to have a derivative that would cross membranes more rapidly than BFT. Indeed, the latter, though often and incorrectly referred to as a hydrophobic molecule, is insoluble in lipids and requires dephosphorylation to the lipid-soluble S-BT (Figure 1) by ectophosphatases. Hence, S-BT would be an obvious candidate, but it is relatively unstable and tends to spontaneously lose the benzoyl group and reform thiamine. Esterification of the thiamine hydroxyethyl chain increases the stability of open thiol forms. In the case of BFT, the presence of a phosphoryl group strongly alters the solubility of the molecule: it is soluble in aqueous solvents at slightly alkaline pH [91].

An alternative would be to replace the phosphoryl group with a more hydrophobic group. An obvious replacement possibility would be the substitution by a second benzoyl group. 

This molecule, O,S-dibenzoylthiamine (DBT, sometimes called bentiamine, Figure 1), is commercially available, but strangely, at the beginning of our studies, only one scientific publication specifically pertaining to the use of this compound in mammals was available [92]. DBT has been known for a long time and is allowed as a food additive in Japan. DBT is composed of a thiamine molecule with an open thiazolium ring linked to two molecules of benzoate, one via a thioester and the other via an O-ester bond. Hence, its conversion to thiamine requires the action of two different enzymes: a thioesterase and an O-esterase [93]. No toxic or tumorigenic side effects have been reported [92], yet there are practically no data about its biological effects. In salmon yearlings, it was better tolerated than thiamine or BFT, and it also led to higher retention of thiamine over time [94].

Hence, this hitherto unexplored derivative could meet the requirements of safety and could be a superior alternative to BFT [93]. Therefore, we decided to test DBT first in cellular models and second in a transgenic mouse model of amyotrophic lateral sclerosis (ALS).

### 8.1. DBT Has Strong Antioxidant and Anti-Inflammatory Properties in Cultured Cells

As already observed for BFT, DBT has strong antioxidant and anti-inflammatory properties, the latter being even more powerful than those of BFT [93]. As shown for BFT, these effects are independent of the coenzyme role of ThDP.

#### 8.1.1. DBT Has Powerful Antioxidant Effects Mediated by Glutathione

At low concentrations (5–10 µM), DBT protects neuroblastoma cells from paraquat toxicity by counteracting oxidative stress [93]. To obtain the same protection with BFT, 5–10-fold higher concentrations are required. DBT increases the synthesis of reduced glutathione and NADPH in an Nrf2-independent manner [93]. Indeed inhibition or knock-down of Nrf2 did not prevent the antioxidant effects of BFT or DBT [93]. A direct stoichiometric effect was also excluded.

#### 8.1.2. DBT Has Powerful Anti-Inflammatory Effects Mediated by NF-κB

DBT has more potent anti-inflammatory properties than BFT. At 10–50 µM, it protects BV2 cells from LPS-induced inflammatory processes (increased expression of iNOS and TNF-α and production of nitric oxide) by translocation of NF-κB into the nucleus [93].

### 8.2. DBT Protects from Motor Neuron Degeneration in a Transgenic Mouse Model of ALS

Chronic administration of DBT (25 mg/kg per day) relieved depressive-like behavior in mice submitted to chronic ultrasound stress [93].

Moreover, DBT arrested motor dysfunction in Fused in Sarcoma (FUS) transgenic mice, a model of amyotrophic lateral sclerosis (ALS). Mutations in the gene encoding the RNA/DNA-binding FUS protein have been detected in familial ALS patients. FUS is a critical component of the oxidative damage repair complex which might explain its role in neurodegeneration. In the FUS [1–359]-transgenic mouse model of ALS, DBT-treated mice displayed improvements in physiological outcomes, motor function, and muscle atrophy compared to vehicle, and the treatment normalized brain levels of GSK-3β, GSK-3β mRNA, and IL-1β mRNA in the spinal cord [17]. 

No clinical data are available on DBT, but a very recent case report showed that BFT positively impacted a patient with ALS [95]. Though a placebo effect cannot be excluded, this study shows that therapy with these derivatives could be useful in patients suffering from ALS.

## 9. Hypotheses on the Mechanisms of Action of Synthetic Thiamine Derivatives

Thiamine prodrugs, and in particular BFT, have been studied at three different levels: (1) cultured cells, (2) rodent models of stress and neurodegenerative diseases, and (3) human patients with mild cognitive impairment or early symptoms of Alzheimer’s disease. These derivatives have strong antioxidant and anti-inflammatory properties, the latter more pronounced for DBT compared to BFT.

**Figure 9:**
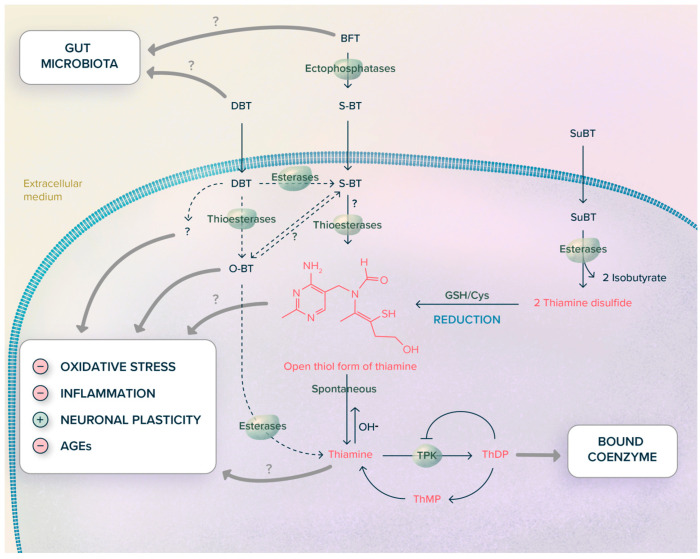
Hypothesis concerning the metabolization of DBT, BFT, and sulbutiamine, based on the data published in the following papers [10,15,32,77,93]. (Figure modified from [12]).

**Figure 10:**
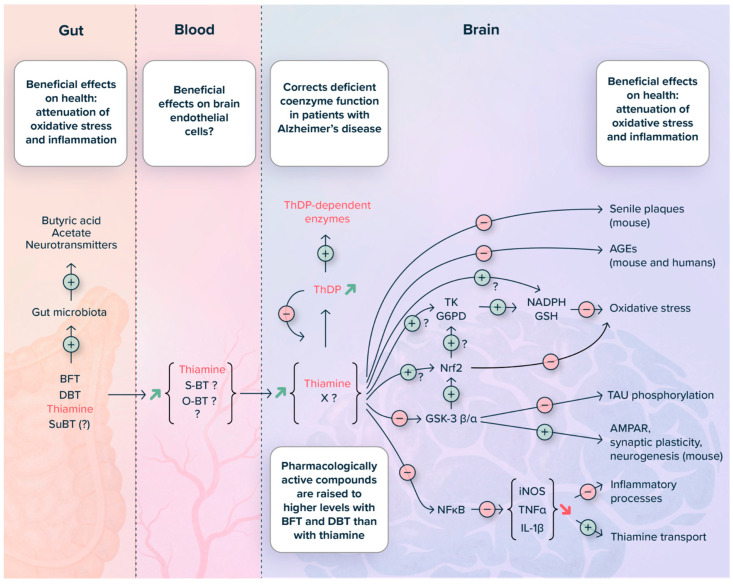
Summary diagram of the pharmacological effects of BFT and DBT. X is a hypothetical intermediate, possibly an open thiol form (Figure 9). (For explanations, see text).

Among the various questions remaining, two are essential for understanding the issues: (1) which is the active metabolite of thiamine prodrugs and (2) which are the molecular targets. Our hypotheses concerning these points are summarized in Figure 9 and Figure 10.

### 9.1. The Pharmacological Effects of BFT and DBT Are Most Probably ThDP-Independent

In thiamine-deficient animals, BFT and DBT, even at low doses, will rapidly restore normal thiamine levels. Pharmacological (mainly antioxidant and anti-inflammatory) effects are observed at high doses (generally above 100 mg/kg). The puzzling, though many times reproduced, observation is that these pharmacological effects in animals are observed while thiamine concentration is only minimally increased in the brain and ThDP concentration not at all.

Unfortunately, this point is ignored in most studies, possibly because the non-coenzyme functions of thiamine are still frowned upon, despite many arguments in their favor [4,96,97,98]. Therefore, we carefully examined this possibility, and our conclusion is that the pharmacological action of DBT and SBT are most probably coenzyme-independent:

1. Most of the studies were conducted in non-deficient cells or animals. Normal chow is rich in thiamine, and it must be understood that thiamine prodrugs are administered under conditions of thiamine saturation, where thiamine-dependent enzymes are saturated with ThDP. Indeed, when tested, ThDP-dependent enzymes are not or, at best, minimally increased [15,93];
2. In animals, administration of thiamine prodrugs does not increase brain ThDP content [15,16,56,75]. This may be linked to feedback inhibition of TPK by ThDP (Figure 6) and, as already mentioned above, is not in favor of an implication of coenzyme function;
3. In cell culture models, the antioxidant and anti-inflammatory effects of thiamine prodrugs persist even when ThDP synthesis is blocked by pyrithiamine [77,93];
4. Many studies claim that BFT activates TK [67,99,100]. It is not clear how this could happen in the absence of a thiamine deficiency as, under normal conditions, TK is close to saturation with ThDP (see point 1). One possibility is that TK expression is upregulated at the mRNA level. Such mechanisms cannot be excluded as TK expression is controlled by Nrf2 [101]. However, using cultured neuroblastoma cells, we observed antioxidant effects of BFT and DBT, though neither TK activity nor expression was increased [93].

These results strongly suggest that ThDP-dependent enzymes are not involved in the pharmacological effects of thiamine prodrugs. Hence, the therapeutic effects of BFT and DBT cannot be reduced to only normalizing coenzyme levels. 

If the actions of thiamine precursors are coenzyme-independent (we prefer coenzyme-independent rather than ThDP-independent as ThDP might have other functions in addition to being a coenzyme), this question is, of course, strongly related to their cellular metabolism responsible for the production of the active metabolite.

### 9.2. If Not ThDP, What Is the Active Metabolite of BFT and DBT?

This question is clearly essential if we aim to understand the action of these compounds and develop better-targeted and more efficient drugs. From the experiments carried out in our laboratory, we can deduce the scheme depicted in Figure 9.

1. BFT requires extracellular dephosphorylation to S-BT (occurring in the intestine in vivo). S-BT, as well as DBT and SuBT, cross plasma membranes by simple diffusion. Intracellular S-BT is subject to the action of thioesterases to yield the open thiol form of thiamine [77]. In an aqueous medium, S-BT spontaneously undergoes an intramolecular rearrangement to O-BT, probably as a result of acyl-migration from sulfur to oxygen [10]. O-BT is then spontaneously converted to thiamine. The conversion of S-BT to O-BT is favored by alkaline pH. Though O-BT can be enzymatically converted to thiamine in most tissues, it does not seem to be more efficient than thiamine in raising tissue thiamine content [102]. O-BT has not been pharmacologically tested, but its spontaneous synthesis from BFT has been reported [10], and docking studies suggested that O-BT might be a good ligand for Keap1, competing for Nrf2 and favoring the translocation of Nrf2 to the nucleus [15]. Therefore, it is not excluded that O-BT might be pharmacologically active. Thiochrome, an oxidized form of thiamine present in small amounts in cells, was not increased by treatment of Neuro2a cells with BFT [77];
2. DBT can either be hydrolyzed to S-BT or yield O-BT through the action of thioesters. O-BT might be transformed to S-BT by acyl migration from an O to an S atom [10]. S-BT can then proceed further to the open thiol form;
3. SuBT, which was not discussed here as it has not been tested in neurodegenerative diseases [63], requires hydrolysis to thiamine disulfide, which must be reduced to the open thiol form. The reductant could be reduced glutathione (GSH) which is the most abundant intracellular thiol [103], or NADPH in a reaction of the type catalyzed by glutathione reductase [104]. SuBT has antioxidant [77] and weak anti-inflammatory [93] properties at a level similar to BFT but, for the latter, did not match DBT;
4. All three thiamine prodrugs considered here will ultimately yield an open thiol form before being processed into thiamine [66]. The formation of the open thiol form from thiamine is unlikely under physiological conditions, but it is a thermodynamically stable form at alkaline pH (pH > 10) [105]. Nevertheless, a challenging hypothesis would be that there exist enzymes able to catalyze the formation of the open form from thiamine [105,106].

The chemistry of the open thiol ring is relatively unexplored. This form is short-lived, but it is possible that in the presence of oxidants (such as G-S-S-G), the open intermediate may react to form -S-S- disulfides with free cysteine thiols of proteins [107]. This might also be an interesting pathway of post-translational protein regulation [107]. Hence, it cannot be excluded that the open thiol form may be pharmacologically active.

Another possibility is that the active compound is simply thiamine formed from the open thiol form, and administration of prodrugs is pharmacologically more efficient than administration of thiamine, because it has a higher impact on intracellular thiamine concentrations. In cultured cells, intracellular thiamine (but not ThDP) concentrations were significantly correlated with cell survival after paraquat poisoning [77].

A further possibility would be that a presently unknown compound is formed from thiamine prodrugs that are stable enough to resist transport from the blood into the brain. So far, neither BFT nor S-BT has been demonstrated to appear in the blood. The absorption of DBT has not been studied, and it is not clear if any reaches the bloodstream. Even if some DBT reaches the bloodstream, it is highly improbable that any would reach the brain parenchyma as it would enter red blood cells and ultimately brain endothelial cells, where it would be transformed into thiamine. Hence, all experimental data suggest that most of the thiamine prodrugs end up as thiamine in the blood.

Though treatment with prodrugs does not lead to increased ThDP levels in the brain, unphosphorylated thiamine is consistently, but not always significantly, increased. Most studies use mouse brain samples of approximately 50–100 mg, which contain all structures, neurons, astrocytes, axons, and synapses. It is possible that increased thiamine levels are limited to very local structures within the sample, but with significant effect. Alternatively, a pharmacologically active compound may be formed from locally increased thiamine.

It is not clear which cell types (endothelial cells, astrocytes of neurons) are involved in this phenomenon. However, brain endothelial cells are in direct contact with blood, the compartment where the highest thiamine increase is observed, and these cells might act as thiamine sensors sending signals to neurons.

### 9.3. Which Are the Targets of BFT and DBT?

As described above, both BFT and DBT have antioxidant and anti-inflammatory effects. In addition, they seem to impact brain plasticity (Figure 10).

1. BFT and DBT (and, to a lesser extent, SuBT and thiamine) have strong antioxidant properties both in vitro [75,77,93] and in vivo [15,17,79]. In cultured neuroblastoma cells, thiamine prodrugs normalized paraquat reduced NADPH and GSH levels in cultured neuroblastoma cells but were independent of TK activity and expression or Nrf2/ARE signaling. These prodrugs clearly seem to be involved in maintaining a high [GSH]/[GSSG] ratio and high NADPH concentrations. These results might be explained by an effect on glucose 6-phosphate dehydrogenase (G6PD). Indeed, this rate-limiting enzyme of the PPP is highly regulated, both at the level of transcription and post-translation [108,109]. However, it must be kept in mind that in addition to the oxidative part of the PPP, other sources of NADPH, such as cytosolic dehydrogenases and mitochondrial trans-hydrogenase, exist [103,109];
2. Decreases in GSK-3β expression or activity regularly turn out after BFT or DBT treatment, in particular in in vivo studies [74,75,76,110] (but [15]). GSK-3β is a pleiotropic serine-threonine protein kinase able to phosphorylate over 100 substrates and play a role in numerous cellular functions affected in Alzheimer’s disease [111]. Decreased activity of GSK-3β activity by phosphorylation on Ser 9 may be responsible for changes in brain plasticity observed [17,74,77]. In addition, GSK-3β might be involved in the antioxidant response by activating Nrf2, an upstream regulator of G6PD and TK [112,113];
3. BFT and, in particular DBT, have strong anti-inflammatory properties that seem to be mediated by NF-κB. Indeed, these compounds block the translocation of its p65 subunit to the nucleus and suppress LPS-induced iNOS expression and cytokine release [79,93]. This is of particular interest in view of the recent results showing that cytokines decrease thiamine transporter expression and activity and that the expression of these transporters is reduced in the brains of Alzheimer’s disease patients [89]. According to our data, DBT is a much more potent anti-inflammatory agent than BFT (and SuBT) in cultured BV-2 cells. We have no explanation for this difference, especially as it does not seem to be more efficient in raising intracellular thiamine concentrations. The differences may be related to different metabolization schemes with different proportions of intermediates O-BT, open thiol form) generated (Figure 9). This might also suggest that antioxidant and anti-inflammatory properties are mediated via different molecular targets;
4. A significant amount of prodrug is already transformed into thiamine in the intestine, thereby affecting the gut microbiome. This might affect the enteric nervous system and, indirectly, the central nervous system. Indeed, thiamine affects the relative abundance of bacterial populations favoring those producing short-chain fatty acids [114]. A very appealing hypothesis would be that BFT corrects gut dysbiosis in Alzheimer’s patients, reducing inflammation and oxidative stress in the brain through the microbiota-gut-brain axis [115,116]. Interestingly NF-κB and Nrf2, both potential targets of BFT, seem to be involved in these processes. However, the gut microbiome hypothesis alone is not sufficient to explain the antioxidant and anti-inflammatory properties of BFT and DBT, as such effects were also observed in cell cultures, suggesting that other mechanisms coexist, as illustrated in Figure 10.

## 10. Conclusions

It is now well documented that human patients with Alzheimer’s disease have impaired thiamine homeostasis, and BFT administrations correct these abnormalities linked to the suboptimal working of ThDP-dependent enzymes [117]. However, we think that in addition to the coenzyme effects, thiamine prodrugs also exert coenzyme-independent effects.

As summarized in Figure 10, these effects are numerous. Some data discussed here were obtained in rodents, while others were obtained in cultured cells. The latter can constitute a simpler model for studying the metabolization of thiamine prodrugs than living animals. However, it is important to be aware of the limitations of cellular models. Indeed, cultured cells are directly exposed to the prodrugs, while brain cells are separated from these molecules by several compartments and barriers (intestinal barrier, blood, and the BBB). 

Hence, the situation is different in animal models, where the prodrugs are administered orally. In the intestine, BFT is dephosphorylated and crosses the intestinal epithelium as S-BT or thiamine. There is so far no experimental evidence that these prodrugs reach the brain as such. Nevertheless, the results obtained with BFT and DBT in cultured cells are consistent with the data obtained in in vivo models, as they clearly show that they have antioxidant and anti-inflammatory properties. Some other studies also showed that BFT prevents the formation of AGEs [15,67]. The exact mechanism and the active derivative involved remain unknown, but all experiments are consistent with effects mediated by GSK-3β and NF-κB.

Considering that there exists no effective treatment for Alzheimer’s disease, we think that thiamine prodrugs have the potential to slow the evolution of the disease. Though only preliminary, present clinical studies show effects of BFT that are at least equal if not superior to monoclonal antibodies directed against ß-amyloid such as Lecanemab or Aduhelm, which are not yet convincing and have important side effects. Thiamine prodrugs have no reported side effects. Anti-tau antibodies do not seem to be effective in patients with early Alzheimer’s disease [118,119]. 

Taken together, those data suggest that both BFT and DBT have a therapeutic potential in brain pathologies associated with oxidative stress and inflammation, not only in neurodegenerative diseases, but also in major depression and aggression linked to stressful events. These effects seem to be independent of the coenzyme role of ThDP.

## Figures and Tables

**Table 1:** Levels of thiamine and ThDP levels in the blood and cerebral cortex of several species.

Species (Tissue)	Thiamine	ThMP	ThDP
**Plasma (nmol/L)**			
Human ^1^	7.1 ± 1.6	5.8 ± 0.6	-
**Blood (nmol/L)**			
Human ^2^	4 ± 3	10 ± 4	138 ± 33
Rat ^3^	188 ± 72	718 ± 90	1127 ± 55
Mouse ^4^	73 ± 10	103 ± 37	737 ± 97
**Cerebral cortex (pmol/mg protein)**			
Human ^2^	0.2 ± 0.3	3.5 ± 2.6	21 ± 5
Baboon (*P. papio*) ^5^	8 ± 2	2.6 ± 0.4	43 ± 7
Rat ^6^	3.1 ± 1.0	4.0 ± 0.4	79 ± 5
Mouse ^4^	6 ± 0.8	8.1 ± 4.6	90 ± 8

^1^ [54], ^2^ [36], ^3^ [55], ^4^ [56], ^5^ modified according to [57], ^6^ [58]. For a more detailed list, see [36,59].

## Data Availability

Not applicable.

## Abbreviations

AGE	Advanced Glycation end products
ALS	Amyotrophic lateral sclerosis
BBB	Blood–brain barrier
BFT	Benfotiamine
DBT	O,S-Dibenzoylthiamine
GSH	Reduced glutathione
G6PD	Glucose 6-phosphate dehydrogenase
O-BT	O-Benzoylthiamine
OGDHC	2-Oxoglutarate dehydrogenase complex
PDHC	Pyruvate dehydrogenase complex
PPP	Pentose phosphate pathway
S-BT	S-Benzoylthiamine
SuBT	Sulbutiamine
ThDP	Thiamine diphosphate
ThMP	Thiamine monophosphate
TK	Transketolase
TPK	Thiamine pyrophosphokinase
